# Stem Cells from Wildtype and Friedreich’s Ataxia Mice Present Similar Neuroprotective Properties in Dorsal Root Ganglia Cells

**DOI:** 10.1371/journal.pone.0062807

**Published:** 2013-05-09

**Authors:** Jonathan Jones, Alicia Estirado, Carolina Redondo, Salvador Martinez

**Affiliations:** Department of Experimental Embryology, Neuroscience Institute-Miguel Hernandez University (UMH-CSIC), Alicante, Spain; National Institutes of Health, United States of America

## Abstract

Many neurodegenerative disorders share a common susceptibility to oxidative stress, including Alzheimer’s, Parkinson Disease, Huntington Disease and Friedreich’s ataxia. In a previous work, we proved that stem cell-conditioned medium increased the survival of cells isolated from Friedreich’s ataxia patients, when submitted to oxidative stress. The aim of the present work is to confirm this same effect in dorsal root ganglia cells isolated from YG8 mice, a mouse model of Friedreich’s ataxia. In this disorder, the neurons of the dorsal root ganglia are the first to degenerate. Also, in this work we cultured mesenchymal stem cells isolated from YG8 mice, in order to compare them with their wildtype counterpart. To this end, dorsal root ganglia primary cultures isolated from YG8 mice were exposed to oxidative stress and cultured with conditioned medium from either wildtype or YG8 stem cells. As a result, the conditioned medium increased the survival of the dorsal root ganglia cells. This coincided with an increase in oxidative stress-related markers and frataxin expression levels. BDNF, NT3 and NT4 trophic factors were detected in the conditioned medium of both wild-type and YG8 stem cells, all which bind to the various neuronal cell types present in the dorsal root ganglia. No differences were observed in the stem cells isolated from wildtype and YG8 mice. The results presented confirm the possibility that autologous stem cell transplantation may be a viable therapeutic approach in protecting dorsal root ganglia neurons of Friedreich’s ataxia patients.

## Introduction

The term oxidative stress is used when there is an imbalance in the cell of reactive oxygen species and the molecules responsible for the removal of these elements. The increased frequency of this condition is known to be related to aging [1], however there are certain diseases where the patients are more susceptible to this condition. These include Alzheimer’s disease, Parkinson, multiple sclerosis, multiple system atrophy, progressive supranuclear atrophy, and Huntington’s disease [2]. Despite their different etiology, these disorders share this common trait in which mitochondrial dysfunction is strongly related [3]. In this respect, iron accumulation due to mitochondrial dysfunction or iron metabolism dysregulation is common in these disorders, which is toxic and ultimately causes cell death [4].

Another disease where oxidative stress plays a major role is Friedreich’s ataxia (FA). This disorder is generally caused by a GAA-triplet repeat expansion in the intron 1 of the frataxin gene [5], [6]. The malfunction of frataxin, which is directly involved in iron homeostasis in the mitochondria, causes iron accumulation similar to that observed in other neurodegenerative diseases [7]. The cells that are most sensitive to this effect are large sensory neurons of the dorsal root ganglia in the spinal cord, the heart (causing cardiomyopathy), and the pancreas [8], [9]. For a review on oxidative stress and its role in FA see [10].

There are currently very few treatments for FA, the majority of which are related to counteract oxidative stress. In this respect, idebenone, a free-radical scavenger, has been shown to have significant results in treating the heart problems associated with the disease in several clinical trials [11]–[15]. Another antioxidant, Coenzyme Q_10_, along with Vitamin E, also gave significant results in both cardiac and skeletal muscle [16]–[18]. However, up to now there is no proof that any of these treatments have positive results in the neurological aspects of the disorder. Thus, it is necessary to develop alternative approaches to treat this aspect of FA.

To this end, our lab studies the possible role stem cells may have in protecting cells susceptible to oxidative stress conditions. Previously we have shown that human adipose stem cell-conditioned medium from healthy individuals protects periodontal ligament cells isolated from FA patients when undergoing oxidative stress [19], which present neural crest stem cell characteristics [20]. The effect observed was due to the presence of various neurotrophic factors in the conditioned medium, which not only upregulated genes implicated in removing reactive oxygen species but also increased frataxin expression. In the present work, we performed a similar experiment, where mouse bone marrow mesenchymal stem cell-conditioned medium was isolated and used in cultured dorsal root ganglia neurons isolated from Fxntm1Mkn/Tg(FXN)YG8Pook (YG8) mice, an FA mouse model. In this case, mesenchymal stem cells were isolated from both healthy and YG8 mice, in order to compare the conditioned medium of these two stem cell populations. The results presented show for the first time that an autologous transplantation of bone marrow stem cells may have a beneficial effect in the protecting the dorsal root ganglia cells of FA patients.

## Materials and Methods

### 1. Animals

All the experiments with animals have been performed in compliance with the Spanish and European Union laws on animal care in experimentation (Council Directive 86/609/EEC), and have been analyzed and approved by the Animal Experimentation Committee of the University Miguel Hernandez and Neuroscience Institute, Alicante, Spain (Reference IN-JJ-001-12). All efforts were made to minimize suffering. Mice were bred and maintained in our animal facilities. Eight month-old Fxntm1Mkn/Tg(FXN)YG8Pook (YG8, originally purchased from Jackson Laboratory) transgenic mice were used. These mice present a knock-out mutation of the Frataxin gene with a human knock-in Frataxin gene to rescue the phenotype [21], [22]. The inserted gene presents the same mutation as in humans, that is, the extended GAA triplet repeats. As a result, the mice present reduced levels of frataxin, causing degeneration in the heart, dorsal root ganglia, and in some cases the pancreas. For the mesenchymal stem cells, the bone marrows of 2–3 month-old C57/BL6 wildtype mice and YG8 mice were used.

### 2. Mesenchymal Stem Cell (MSCs) Extraction and Culture

The protocol used was similar to our previously published work [23]. Briefly, femurs were dissected from 2–3 month old mice, sacrificed by cervical dislocation. Bone marrow was extracted, and single-cell suspensions were obtained by mechanical dissociation. Then, the suspension was washed and centrifuged, and the pellet re-suspended in D-MEM (Invitrogen) supplemented with 15% FBS (Biochrom AG, Berlin), and 100 U/ml penicillin/streptomycin (Sigma-Aldrich). These cells were placed in culture flasks and the plastic-adherent population was isolated and allowed to proliferate for 3–4 weeks, changing the media every 2–3 days and replating when needed.

### 3. Dorsal Root Ganglia Isolation and Culture

Dorsal root ganglia from YG8 mice were extracted and cultured as described previously [24]. The dorsal root ganglia of the whole spinal cord were isolated and disaggregated in 1 mg/ml collagenase type XI (Sigma) and 3 mg/ml dispase (Gibco) and cultured in Dulbecco's modified Eagle's medium/F-12 medium, containing 10% fetal bovine serum (Invitrogen), and supplemented with 4 mm l-glutamine (Invitrogen), 17 mm glucose, nerve growth factor (100 ng/ml; Sigma), and antibiotics. Cells were plated on either on poly-lysine coated glass coverslips or directly on poly-lysine treated multiwell plates. Twenty-four hours after extracting the dorsal root ganglia, the medium was changed to D-MEM supplemented with 15% FBS and penicillin/streptomycin, the same medium used to culture the MSCs. Then, after an additional 24 hours, the cells were cultured in one of the culture conditions commented in the next section. In several cases, the kinase inhibitor K252a (Sigma-Aldrich) was used, in order to confirm if NT3, NT4 and BDNF trophic factors that are present in the conditioned media were responsible for the observed effects. To this end, the cells were first incubated for 1 hour with 200 nM of K252a, then the medium was changed to the corresponding conditioned medium with K252a (200 nM) and H2O2 for an additional 24 hours.

### 4. Hydrogen Peroxide Treatment

The procedure used was similar to our previous work [19]. The dorsal root ganglia cells were cultured in standard medium (D-MEM plus FBS and antibiotics) and supplemented with 0.1 mM hydrogen peroxide (Sigma-Aldrich) for 24 hours. Hydrogen peroxide was added at the beginning of the experiment (two days after DRG extraction), and 24 hours afterwards the cells were collected and processed accordingly (see below). The dorsal root ganglia cells were submitted to one of four culture conditions: under normal culture conditions (Control), exposed to hydrogen peroxide (H2O2) and finally exposed to hydrogen peroxide and cultured in conditioned medium from wildtype or YG8 stem cells (MSC-WT and MSC-AtF, respectively).

To collect the conditioned media of wildtype and YG8 stem cells, this was recovered 24 hours after incubation under normal culture conditions. In several cases, conditioned medium from stem cells exposed to hydrogen peroxide for 24 hours was recovered. Four different batches of conditioned medium were isolated from wildtype and YG8 stem cells each. These batches were obtained from stem cells isolated from mice of the same age and cultured the same time period. The conditioned media were centrifuged at 1500 rpm for 10 minutes and the resulting pellet discarded.

### 5. Quantitative PCR

Total mRNA of the cells was isolated using the Trizol protocol (Invitrogen). Five micrograms of mRNA was reverse-transcribed, and approximately 100 ng of cDNA was amplified by Real Time PCR using Power SYBR Green Master mix (Applied Biosystems). All the samples were run in triplicate using the StepOne Plus Real-Time PCR system (Applied Biosystems) and analyzed with the StepOne Software. Analyses were carried out using the delta C(T) method and calculated relative to GAPDH (forward: AGGTCGGTGTGAACGGATTTG, reverse: GGGGTCGTTGATGGCAACA). The results were normalized with respect to the control condition, which presented a value of 1, using the same approach as in our previous report [19]. The following primers were used, taken from the PrimerBank webpage (http://pga.mgh.harvard.edu/primerbank/): GDNF (forward: CGCCGGTAAGAGGCTTCTC, reverse: CGTCATCAAACTGGTCAGGATAA), NT4 (forward: TGAGCTGGCAGTATGCGAC, reverse: CAGCGCGTCTCGAAGAAGT), NGF (forward: GCACTACACCCATCAAGTTCA, reverse: TCCTGAGTCATGCTCACAAGT), BDNF (forward: TCATACTTCGGTTGCATGAAGG, reverse: GTCCGTGGACGTTTACTTCTTT), NT3 (forward: AGTTTGCCGGAAGACTCTCTC, reverse: GGGTGCTCTGGTAATTTTCCTTA), Frataxin (forward: CCACGCCCATTTGAACCTC, reverse: TCTTTCATACGCTGTCTCGTCT), SOD1 (forward: ATGGCGATGAAAGCGGTGT, reverse: CCTTGTGTATTGTCCCCATACTG), SOD2 (forward: CAGACCTGCCTTACGACTATGG, reverse: CTCGGTGGCGTTGAGATTGTT), SOD3 (forward: CCTTCTTGTTCTACGGCTTGC, reverse: ACGTGTCGCCTATCTTCTCAA), Caspase-3 (forward: TGGTGATGAAGGGGTCATTTATG, reverse: TTCGGCTTTCCAGTCAGACTC), Catalase (forward: AGCGACCAGATGAAGCAGTG, reverse: TCCGCTCTCTGTCAAAGTGTG), GPX-1 (forward: CCACCGTGTATGCCTTCTCC, reverse: AGAGAGACGCGACATTCTCAAT), BCL-2 (forward: ATGCCTTTGTGGAACTATATGGC, reverse: GGTATGCACCCAGAGTGATGC), Bax (forward: AGACAGGGGCCTTTTTGCTAC, reverse: AATTCGCCGGAGACACTCG).

### 6. Western Blot

The lysis buffer and process used was similar to that of [19]. Cell lysates and culture media were separated on 15% SDS-polyacrylamide gels, and probed for rabbit anti-Frataxin in the case of cell lysates (1∶750, Santa Cruz Biotechnology), while mouse anti-Tuj1 (1∶1000, Covance), rat anti-GFAP (1∶1000, Calbiochem), rabbit anti-BDNF (1∶500, Santa Cruz Biotechnology), anti-NT3 (1∶250, Abcam) and anti-NT4 (1∶250, Santa Cruz Biotechnology) were analyzed in the culture media. Secondary antibodies were visualized by chemiluminescence (ECL, Amersham). For protein quantification we used Quantity One software (BioRad). Beta-actin was used as loading control, except where conditioned medium was studied, in which case Ponceau S (Sigma-Aldrich) staining was used.

### 7. Immunocytochemistry

A standard immunocytochemistry protocol was used, similar to previous studies [23]. Primary antibodies used were mouse anti-Tuj1 (1∶1000, Covance), rat anti-GFAP (1∶500, Calbiochem), and DAPI as nuclear staining. As for secondary antibodies, Alexa Green (1∶500, Molecular Probes) was used for GFAP, and for Tuj1 staining (in red), biotinylated mouse secondary antibody was used (1∶200, Vector Laboratories, Burmingham, California) followed by an incubation with streptavidin conjugated with Cy3 (1∶500). Samples were visualized and images taken with a Leica fluorescence microscope (Leica DMR, Leica Microsystems).

### 8. Statistical Analysis

Statistical significance between control and experimental groups were calculated with Sigmaplot v11.0 software, using the paired t-test and non-parametric (Kruskal-Wallis) test where applicable, establishing the level of significance at p<0.05. Values are measured as mean +/− standard deviation.

## Results

### 1. Mesenchymal Stem Cell-conditioned Medium Increased Cell Survival in Dorsal Root Ganglia Cells of Friedreich’s Ataxia Mice

**Figure 1:**
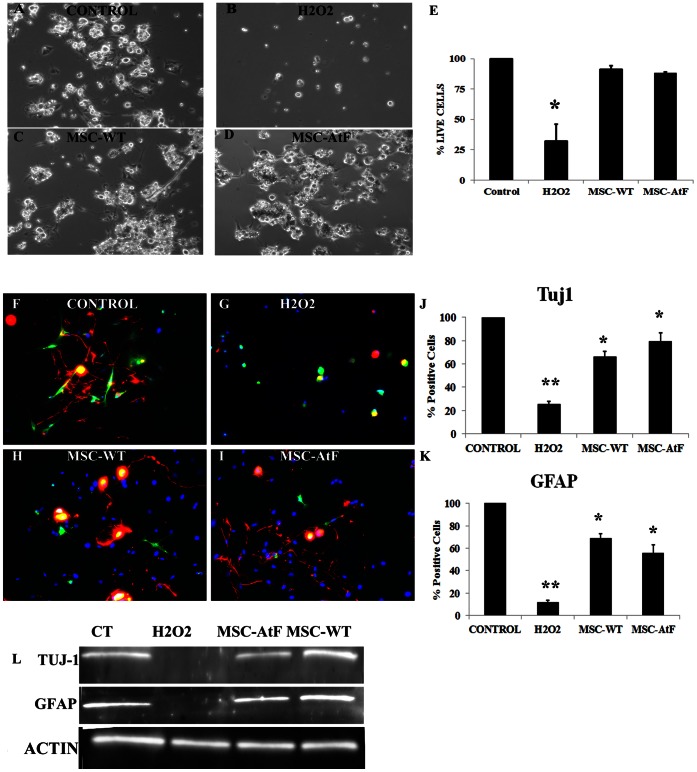
Dorsal root ganglia cultures exposed to hydrogen peroxide. (A–D) Dorsal root ganglia primary cultures before (A), 24 hours after exposure to hydrogen peroxide (B), and 24 hours after exposure to hydrogen peroxide and cultured with conditioned medium from wildtype and YG8 stem cells (C and D, respectively). E) The histogram depicts the percentage of live cells in culture among the different culture conditions, considering the control culture as 100%. After being submitted to oxidative stress (labeled H2O2 in the histogram), over 67% of the cells in culture die. However, almost all cells survive when exposed to hydrogen peroxide and under mesenchymal stem cell-conditioned medium. F–I) Immunocytochemistry of Tuj1 (in red) and GFAP (in green) of the dorsal root ganglia cultures. J–K) The histograms present the percentage of cells positive for Tuj1 and GFAP, respectively, in the different cell cultures. The percentages are with respect to the control, normalized to 100%. L) Western blot analysis of Tuj1 and GFAP in the various culture conditions, with beta-actin as control. N = 3 in all cases, *p<0.05, **p<0.01.

Dorsal root ganglia cells isolated from YG8 mice were cultured under different conditions: 1) standard culture medium (DMEM plus FBS and penicillin/streptomycin), 2) exposed to H2O2, and H2O2 with conditioned medium isolated from mesenchymal stem cells of wildtype (MSC-WT, 3) or YG8 mice (MSC-AtF, 4) (Figure 1 A–D, respectively). Dorsal root ganglia cells cultured with conditioned medium (without H2O2) did not present any morphological changes or cell death compared to the cells under standard culture medium (data not shown). The same number of cells was placed in each condition. After 24 hours of culture, only 32% +/−14% of the cells remained alive when exposed to H2O2 and without the conditioned medium (Figure 1B and E). The neurons, which originally were elongated and presented one or various branches (Figure 1A), either died and detached from the plate or remained alive, albeit losing their original morphology (Figure 1B).

On the other hand, almost all cells survived when they were exposed to H2O2 and cultured in mesenchymal stem cell-conditioned medium from wildtype or YG8 stem cells (Figure 1C and D, respectively). The surviving cells maintained their original morphology and seemed to be unaffected by the oxidative stress.

DRG cultures contain multiple cell types, including neurons, satellite cells, Schwann cells, and fibroblasts. Thus, to analyze what cell types were more susceptible to oxidative stress, we performed immunocytochemistry of the cultures for Tuj1 and GFAP (Figure 1F–I), which are neuronal and Schwann/satellite cell markers, respectively. Although myelinating Schwann cells do not express GFAP in vivo, in vitro they de-differentiate into immature (non-myelinating) Schwann cells, which do express GFAP [25]. As a result, in the DRG cultures exposed to H2O2 there was a 80–90% neuronal and satellite/Schwann cell loss compared to control conditions (Figures 1G, J and K). Meanwhile, in the cultures with the conditioned medium (Figures 1H–K), there was little cell loss. Furthermore, the neurons in these cultures presented a similar morphology as those observed in the control culture condition, with long axonal prolongations, whereas the H2O2 condition lost their axons. Also, the Schwann cells, generally seen as thin, elongated cells (Figure 1F), were reduced in size and round, as if undergoing cell death (Figure 1G).

The results observed by immunocytochemistry were corroborated by Western blot analysis (Figure 1L). In the H2O2 cultures there was no visible band of either Tuj1 or GFAP, while the DRG cells with the conditioned media presented similar bands as those observed in the control condition. The lack of Tuj1 and GFAP expression in the Western blot indicated that the number of surviving cells was too low in the H2O2 condition to be detected by this technique. No differences were observed in the number and cell types that survived when comparing the conditioned medium of wildtype and YG8 stem cells.

Thus, mesenchymal stem cell-conditioned medium increases the cell survival of dorsal root ganglia cells when exposed to hydrogen peroxide.

### 2. MSC-conditioned Medium Increased Frataxin Expression as Well as Oxidative Stress and Pro-Survival Markers in Dorsal Root Ganglia Cells

**Figure 2:**
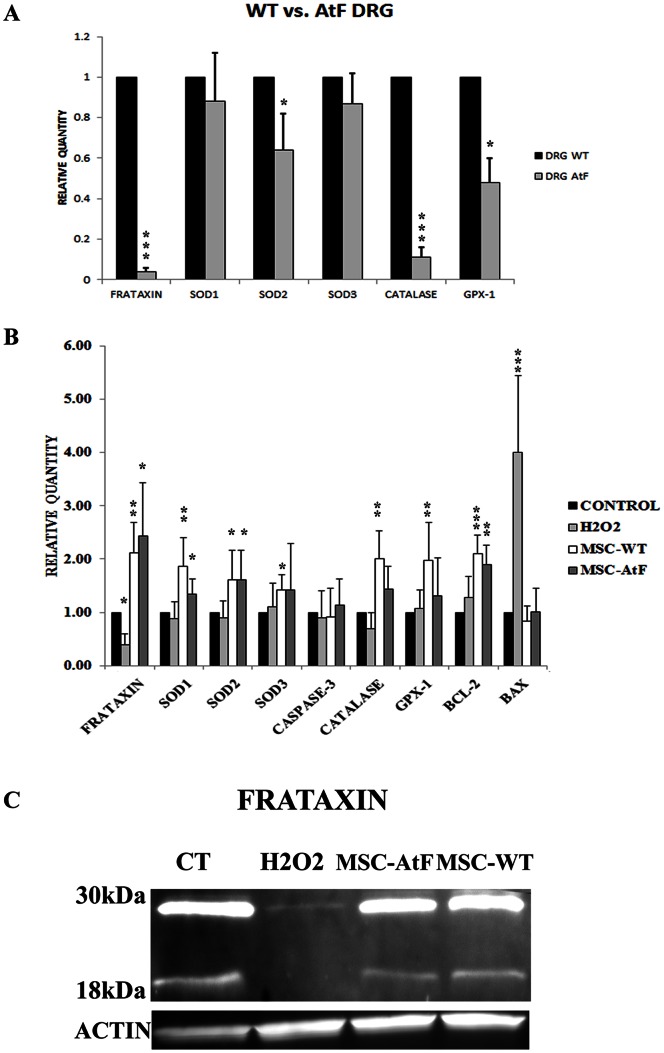
Quantitative analysis of dorsal root ganglia cells. A) Quantitative PCR analysis of dorsal root ganglia cells of wildtype (WT) versus YG8 (AtF) mice under normal culture conditions. B) Quantitative PCR analysis of dorsal root ganglia cells under normal culture medium (Control), exposed to hydrogen peroxide (H2O2) and exposed to hydrogen peroxide with conditioned medium from wildtype and YG8 stem cells (MSC-WT and MSC-AtF, respectively). H2O2 cultures express high Bax levels, indicating apoptosis of surviving cells. DRG cultured with conditioned medium presented a significant increase in the expression levels of frataxin, oxidative stress markers, as well as high Bcl-2 and low Bax levels. N = 6 in all cases except for MSC-AtF where n = 4, *p<0.05, **p<0.01, ***p<0.001. C) Western blot analysis of frataxin in the various culture conditions (n = 4). Two bands were observed, one at 30 kDa corresponding to the precursor form of frataxin, and one at 18 kDa corresponding to the mature form.

Before analyzing the DRG from YG8 mice, these were isolated and compared under normal culture conditions with their wildtype counterparts to study the expression of frataxin as well as oxidative stress-related genes (Figure 2A). First of all, frataxin expression was over 10 times lower in the DRGs of YG8 mice, as expected. Furthermore, lower levels of expression of various oxidative stress markers were detected in the YG8 DRGs, including SOD2, catalase and GPX-1. Thus, under normal conditions, the DRGs of ataxic mice present lower expression levels of genes that eliminate ROS elements of the cell. This was also observed in our previous report using cultured cells isolated from FA patients and compared with healthy individuals [19].

These same genes, as well as cell survival and apoptosis markers, were analyzed in the various culture conditions in order to elucidate the possible mechanisms for the increased cell survival in the cells cultured in mesenchymal stem cell-conditioned medium and under oxidative stress. Frataxin expression decreased when dorsal root ganglia cells were exposed to hydrogen peroxide (less than half of the expression detected in normal conditions, Figure 2B). However, under mesenchymal stem cell-conditioned medium, frataxin was increased 4-fold compared to the cells exposed to H2O2 (and almost 2-fold compared to normal culture conditions). Similar results were observed when mesenchymal stem cell-conditioned medium from YG8 stem cells was used. Frataxin expression was also studied by Western blot analysis (Figure 2C). Two bands were detected, one at 30 kDa corresponding to the precursor form, and the mature form at 18 kDa [26]. The results of the western blot showed that frataxin levels were greatly decreased in hydrogen peroxide-exposed dorsal root ganglia cells, where only a slight band of the precursor form was detected, while mesenchymal stem cell-conditioned medium restored its expression to normal levels.

The transcription levels of genes implicated in oxidative stress were also analyzed by quantitative PCR, such as SODs 1–3, catalase and GPX-1 (Figure 2B). In this case, almost all oxidative stress markers significantly increased when the DRG cells exposed to hydrogen peroxide were cultured in stem cell conditioned medium, independently if the medium derived from wild-type or YG8 stem cells. This increased expression was not observed in the cells exposed to hydrogen peroxide.

As for apoptosis markers, caspase-3 expression did not differ in any of the culture conditions. Caspase-3 is activated under certain circumstances when the cell undergoes apoptosis. However, it is generally a rapid process and is possible that after 24 hours of hydrogen peroxide exposure, caspase-3 may have been activated and the affected cells have died, while the remaining cells were capable of withstanding the oxidative stress assault. In the latter case, caspase-3 expression would remain unchanged compared to control conditions. On the other hand, Bcl-2 and Bax levels, which are also survival and apoptosis markers respectively, differed. Under oxidative stress, the dorsal root ganglia cells exposed to hydrogen peroxide expressed slightly but not significantly higher levels of Bcl-2 (cell survival marker) than control conditions, while Bax (apoptosis marker) was increased almost 4-fold. This indicates that there was an induction towards apoptosis-mediated cell death in the surviving cells after incubation with hydrogen peroxide. As for the cells cultured in mesenchymal stem cell-conditioned medium, Bcl-2 expression was increased over 2-fold while Bax levels were strongly reduced compared to the cells exposed to hydrogen peroxide, maintaining similar expression levels as the controls. This indicated that pro-apoptotic signals were being blocked due to the stem cell conditioned medium, in favor of survival markers such as Bcl-2. As in other cases, there were no significant differences in the expression levels of the analyzed genes when using stem cell conditioned medium from wild-type or YG8 stem cells.

### 3. Mesenchymal Stem Cells Secrete Trophic Factors NT3, NT4, and BDNF

**Figure 3:**
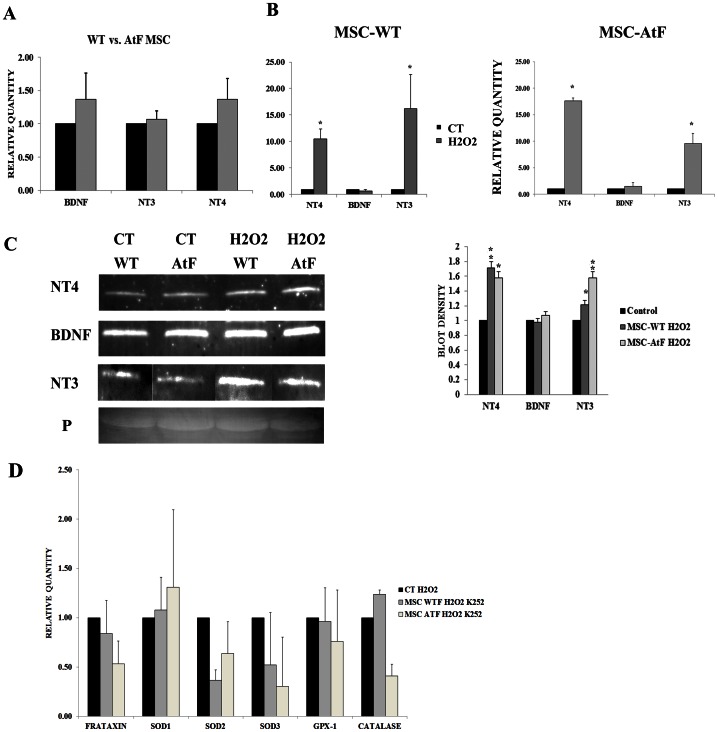
Quantitative analysis of trophic factors BDNF, NT3 and NT4 in mesenchymal stem cells and MSC-conditioned medium. A) Quantitative PCR analysis of trophic factors released in the conditioned medium of mesenchymal stem cells isolated from wildtype (WT) and Friedreich’s ataxia mice (AtF) under normal culture conditions. B) Quantitative PCR analysis of trophic factors released in the conditioned medium of mesenchymal stem cells isolated from wildtype (MSC-WT) and Friedreich’s ataxia mice (MSC-AtF), under normal culture conditions (CT) and exposed for 24 hours to hydrogen peroxide (H2O2). N = 6, *p<0.001. C) Western blot analysis of the trophic factors in mesenchymal stem cell-conditioned medium under normal culture conditions and exposed to hydrogen peroxide. P indicates Ponceau S staining as loading control. To the right, gel density analysis of the western blots, where hydrogen peroxide treated stem cells (MSC-WT H2O2 and MSC-AtF H2O2) were compared to their respective control culture condition. N = 4 in all cases, *p<0.05, **p<0.01. D) Quantitative PCR analysis of oxidative stress-related genes, as well as frataxin, of dorsal root ganglia cultures exposed to oxidative stress (CT H2O2) as well as with the stem cell conditioned medium of wildtype (MSC-WT H2O2 K252) and YG8 (MSC ATF H2O2 K252) mice.

There are 3 main sensory neurons in the dorsal root ganglia, nociceptive/thermoreceptive, mechanoreceptive and proprioceptive. The nociceptive/thermoreceptive neurons express TrkA receptors that respond to NGF. Mechanoreceptive neurons express the TrkB receptor and respond to BDNF and NT4. Finally, proprioceptive neurons, the first to degenerate in Friedreich’s ataxia and in YG8 mice, express the TrkC receptor and respond to NT3. We decided to analyze these trophic factors by quantitative PCR and Western blot in the mesenchymal stem cells from wildtype and YG8 mice as well as their conditioned medium. First, the conditioned medium from wildtype and YG8 stem cells were analyzed for the trophic factors NT4, NT3 and BDNF under normal culture conditions (Figure 3A). NGF and GDNF expression was note detected (data not shown). No significant differences in the expression of these trophic factors were detected comparing wildtype and YG8 mesenchymal stem cells.

Also, we studied the expression levels of these factors in response to oxidative stress (Figure 3B), in order to analyze if the stem cells respond to this stress by increasing the expression of the trophic factors. As a result, both NT3 and NT4 increased their gene expression levels over 10-fold when the cells were exposed to oxidative stress, while BDNF levels remained the same. This was observed both in wildtype and YG8 stem cells. Western blot analysis of the conditioned medium confirmed that the stem cells secreted higher levels of NT3 and NT4 when exposed to hydrogen peroxide (Figure 3B and C). However, BDNF expression, while it did not increase in response to the oxidative stress, presented a very strong band of expression compared to the other neurotrophic factors.

Finally, to confirm if the trophic factors analyzed were responsible for at least part of the observed effect of the conditioned media, the DRG cells were co-cultured with K252a, a known kinase inhibitor. This protein blocks TrkA, TrkB and TrkC receptors, thus inhibiting the effect of the trophic factors analyzed. As a result, the expression of the oxidative stress-related genes analyzed in the cultures with conditioned medium did not differ from the control condition (Figure 3D). DRG cultures with K252a and under normal culture conditions were not affected by the kinase inhibitor (data not shown). Thus, the blockage of these kinase receptors effectively inhibits the upregulation of oxidative stress-related genes observed when the DRGs were cultured with stem cell-conditioned medium, confirming that the trophic factors NT3, NT4 and BDNF play a major role in the observed effect.

## Discussion

In this work, we demonstrated that bone marrow mesenchymal stem cells from a Friedreich’s ataxia (FA) mouse model, presented similar neurotrophic properties than their wild-type counterpart. Furthermore, conditioned medium isolated from the stem cells was capable of increasing the cell survival of neurons isolated from the mouse model when exposed to oxidative stress.

As indicated in the results section, we analyzed the neurotrophic factors BDNF, NT3 and NT4 in the conditioned medium of stem cells isolated from wildtype and YG8 mice. These neurotrophic factors bind to different neuronal populations of the dorsal root ganglia, being the proprioceptive neurons the first to degenerate in FA [27]. Proprioceptive neurons express TrkC membrane receptor, which responds to NT3 [28]. By binding to their receptors, the neurotrophic factors activate several mechanisms, including cell survival and proliferation, or even regeneration of peripheral nerves [29]. Both YG8 and wild-type bone marrow stem cell conditioned medium increased survival markers in the dorsal root ganglia cells cultured with hydrogen peroxide, as well as decreased apoptosis and activated the transcription of certain oxidative stress-related genes. The presence of these trophic factors in the conditioned medium indicates a possible role of these factors in the survival of the cultured dorsal root ganglia cells under oxidative stress.

Throughout the lifespan of an individual, his/her cells are exposed to oxidative stress, which is generally removed due to an efficient antioxidant system. However, in the case of FA, the necessary mechanisms to remove the ROS are deficient. This causes early cell death which spills out additional ROS elements to the nearby cells, causing oxidative stress to them and further damage. All this is due, in part, to the lack of frataxin, which causes iron overload and increased free-radical production [30]. Our data demonstrate that MSCs, if transplanted near cells susceptible to this condition, can respond to the oxidative stress by releasing factors that increase the survival rate of the affected cells due to the upregulation of ROS-related genes. Furthermore, when the MSCs were cultured under oxidative stress, this condition activated mechanisms to increase the expression and secretion of these trophic factors.

Even though proprioceptive neurons are the first to degenerate in Friedreich’s ataxia, ultimately all the neurons of the dorsal root ganglia, as well as Schwann cells, are affected [31]. In our study, we decided to isolate dorsal root ganglia from 8 month-old mice, as previous works indicated that degeneration of the neurons in this region can be detected at 6–12 months of age [22]. In this manner, we may study if stem cell condition medium may help avoid the degeneration of a heterogeneous population of cells.

As indicated in previous works, the unstable GAA repeat expansions in FA produces gene transcriptional reduction or silencing of frataxin [32], most likely due to epigenetic processes [6], [33]–[36]. Thus, it is possible to consider that reversal of frataxin gene silencing may be achieved through epigenetic-modifying compounds, and considered as a potential therapeutic approach. To this end, neurotrophic factors are capable of reversing to some extent this gene silencing, which has been proven both in our previous publication [19] as well as in this current work. Specifically, in our previous work we demonstrated that BDNF was capable of increasing frataxin expression, as well as oxidative stress-related markers and cell survival, when FA cells were exposed to oxidative stress. The potential beneficial effect of BDNF in ataxia has been previously proven in an ataxic mouse model, where inoculation of BDNF transgene into stargazer mutant mice improved their behavior test [37]. As mesenchymal stem cells express a number of trophic factors, it is plausible to consider that this same neuroprotection detected in our previous work can be extrapolated to a multiple cell type structure such as the dorsal root ganglia.

In conclusion, this work demonstrates for the first time that mesenchymal stem cells from diseased mice present similar neuroprotective properties as those observed in wildtype mice. This observation indicates the possibility that autologous stem cell transplantation in FA patients may be feasible in order to protect their affected neurons. Further studies in this respect may help in a near future towards a stem cell-based therapeutic application in this affected population. Finally, as oxidative stress is an important issue in various neurodegenerative diseases, the information collected in this work can be extrapolated to these disorders, which include Alzheimer’s, Parkinson, and Huntington disease.
